# Knowledge and Attitudes Regarding Cataracts and Their Associated Factors Among Hail Region Residents in Saudi Arabia

**DOI:** 10.7759/cureus.60444

**Published:** 2024-05-16

**Authors:** Reema S Alanazi, Areeb F Alshammari, Fatima H Albladi, Atheer Alanizy, Abrar Ali, Nabeel Shalabi

**Affiliations:** 1 College of Medicine, Hail University, Hail, SAU; 2 Ophthalmology, Hail University, Hail, SAU

**Keywords:** saudi arabia, hail, knowledge, attitudes, cataract

## Abstract

Background

Cataract is a condition that affects the lens, causing separation and/or aggregation of proteins and disrupting the regular alignment of cell fibers. Cataracts have many known risk factors contributing to their development, such as diabetes, oral steroid therapy, smoking, and high body mass index. Good knowledge about cataracts may improve the quality of treatment through early diagnosis. Unfortunately, poor knowledge is still a significant barrier to reducing blindness caused by cataracts in developing countries.

Methodology

This cross-sectional study was conducted using a pre-validated questionnaire and online questionnaires. Participants were approached through multiple social media platforms from June 2022 to August 2022.

Results

Of the 307 participants, with a mean age of 32.4 ± 12.8 years, 51.5% had good knowledge of cataracts and their related risk factors, while 28.7% had a favorable attitude about cataracts. Of all participants, 50.5% reported a diagnosis of cataract. The majority of participants, 58.6%, who did not have cataracts, had good knowledge versus 44.5% of others with (p = 0.014). Moreover, 75% of participants aged 50 years or older had good overall cataract knowledge levels compared to 38.9% of others aged 30-39 years (p = 0.002).

Conclusions

Cataracts are a preventable cause of blindness that can be corrected by surgery. In this study, we discovered extremely concerning rates of knowledge, awareness, and attitudes regarding cataracts among the populations of Hail City. More educational programs should be directed toward spreading knowledge about cataracts to patients and the public.

## Introduction

Cataract affects the lens, causing the usually transparent lens to cloud. Depending on the location of the clouding, it can affect vision to varying degrees. It is believed that the most plausible cause for lens cloudiness is the separation and aggregation of proteins; another reason is the disruption of the regular alignment of cell fibers [1]. According to the World Health Organization, cataracts are one of the leading causes of blindness worldwide, causing 94 million people to have moderate or severe distance vision impairment or blindness [2]. In a Saudi study, the prevalence of visual impairment was 13.9% (95% confidence interval = 11.4%-16.9%), and cataract was the underlying cause for 29.1% of cases [3].

Many risk factors play a role in the development of cataracts, such as diabetes, oral steroid therapy, current smoking, high body mass index, occupational exposure to sunlight, gout medications, family history, and use of eyeglasses by age 20 years [4]. According to the Ministry of Health, the most reported symptoms of cataracts are gradual and painless weakening of vision, sensitivity to bright lights, formation of halos around lights, struggle to see clearly at night, and the lens gradually changing its color to white or brown as cataract advances [5]. The public needs to be wary of these symptoms and seek ophthalmologist advice. Regarding cataract intervention, cataract surgery has developed into a small-incisional procedure with quick vision recovery, positive visual outcomes, and few complications in most patients because of advancements in surgical technology and techniques. The treatment of cataracts with astigmatism, presbyopia, or both is now achievable because of advances in intraocular lens technology [6]. As a variation of extracapsular cataract surgery, manual small-incision cataract surgery also has similar intraoperative and postoperative problems, according to studies on its effectiveness and safety for cataract surgery. Therefore, the risk of iris damage, striate keratitis, and posterior capsular rupture is increased by the significant handling that occurs inside the anterior chamber during nucleus administration [7]. In addition to the complication of cataract surgery, posterior-capsule opacification is considered a primary cataract surgery complication that occurs the most frequently [8]; hence, the rectangular, sharp-edged optic design, which produces a sharp capsular bend, is the fundamental cause of the effectiveness of the sharp-edge foldable intraocular lens types in preventing posterior capsule opacification [9].

A cataract is a condition influenced by an individual’s occupation, lifestyle, and general health. Therefore, awareness should be raised, and the public should be educated. Multiple studies conducted in different regions in Saudi Arabia have shown poor knowledge and understanding regarding cataracts [10-13]; however, none of these studies assessed the awareness and knowledge of the Hail population. Therefore, conducting such research in the Hail region would be needed to understand and measure the public’s awareness of cataracts and their factors.

Objectives

This study aimed to explore the knowledge, attitudes, and awareness of Hail region residents regarding cataracts and their causes, associated risk factors, and treatment options.

## Materials and methods

Study design

This cross-sectional study was conducted using a validated questionnaire after the approval of the Institutional Review Board (approval number: H-2022-273). A total of 307 participants aged 18 and above and residing in the Hail region were included. Participants were recruited using an electronic online questionnaire through multiple social media platforms (e.g., WhatsApp, Twitter, Telegram) from June 2022 to August 2022 in the Hail region of Saudi Arabia. The agreement to participate in the study was considered proof of consent. The demographic data of the participants were recorded and analyzed.

Content of the survey instrument

A 24-item questionnaire was developed to assess the knowledge, attitudes, and awareness of cataracts and their associated risk factors in the Hail region. Initially, items one through six were about participants’ data. Participants’ knowledge about cataracts and their consequences was tested using 15 items. One item was used to assess participants’ source of information and where they received their knowledge of cataracts. Finally, five items were designed to assess participants’ attitudes and awareness regarding cataracts.

Sample size

In total, 307 participants were needed to estimate the knowledge, attitudes, and awareness of cataracts and their associated risk factors in the Hail region. The sample size calculated for this cross-sectional study used the following formula = Z 1-ᾳ/2 2 SD2/d2, where Z1-α/2 is the standard normal variate (5% type 1 error) and a confidence level of 95%. SD is the standard deviation of the variable and d is the absolute precision.

Statistical analysis

The data were collected, reviewed, and fed to SPSS version 21 (IBM Corp., Armonk, NY, USA). All statistical methods used were two-tailed with an alpha level of 0.05, considering significance if the p-value was less than or equal to 0.05. A descriptive analysis was done, and the knowledge and attitude of adult participants regarding cataracts, risk factors, and treatment were tabulated. Participants’ awareness regarding cataract prevention and their source of information on cataracts were graphed. Pearson chi-square test for significance and exact probability test were used to determine small frequency distributions in participants’ overall knowledge level by their data and source of information, as well as testing the distribution of participants’ attitudes by gender using the latter test.

Overall knowledge level regarding cataracts was assessed by summing up discrete scores for multiple correct knowledge items. The general knowledge score was categorized as poor if the participant’s score was less than 60% of the overall score and good if the participant’s score was 60% or more.

## Results

As shown in Table 1, a total of 307 adults completed the study questionnaire. Participants’ ages ranged from 20-50 years, with a mean age of 32.4 ± 12.8 years. Overall, 169 (55%) participants were females, and 155 (50.5%) were married. Regarding educational level, 187 (60.9%) participants were university graduates, while 94 (30.6%) had secondary education or below. A total of 110 (35.8%) participants were students, 140 (45.6%) were non-healthcare workers, and 40 (13%) were healthcare workers. In total, 155 (50.5%) had cataracts.

**Table 1:** Demographic data of study participants, Hail, Saudi Arabia.

Demographic data	N	%
Age in years		
20–29	117	38.1%
30–39	126	41.0%
40–49	52	16.9%
50+	12	3.9%
Gender		
Male	138	45.0%
Female	169	55.0%
Marital status		
Single	152	49.5%
Married	155	50.5%
Educational level		
Secondary/Below	94	30.6%
University	187	60.9%
Postgraduate	26	8.5%
Work		
Not working/Retired	17	5.5%
Student	110	35.8%
Non-healthcare staff	140	45.6%
Healthcare staff	40	13.0%
Have you been diagnosed with cataracts		
Yes	155	50.5%
No	152	49.5%

A total of 112 (36.5%) study participants reported that cataracts are lens changes where the lens becomes opaque, while 58 (18.9%) said that cataracts are an age-related process leading to a decrease in vision. Moreover, 238 (77.5%) knew that cataracts decreased vision and 176 (57.3%) said that cataracts cause blindness, whereas 208 (67.8%) knew that it is possible to regain vision caused by cataract blindness. Considering cataract risk factors, the most known among study participants were hypertension at 198 (64.5%), followed by old age at 197 (64.2%), diabetes mellitus at 192 (62.5%), trauma at 189 (61.6%), ultraviolet light at 186 (60.6%), and obesity at 181 (59%). Regarding cataract treatment, 157 (51.1%) study participants chose surgery over medications, while 187 (60.9%) thought that it was necessary to implant a lens in cataract surgery (Table 2).

**Table 2:** Knowledge of cataracts, consequences, risk factors, and treatment, Hail, Saudi Arabia.

Domain	Items	N	%
General knowledge of cataracts and consequences	What is cataracts		
	A lens changes where it becomes opaque	112	36.5%
	An age-related process leading to a decrease in vision	58	18.9%
	A white membrane growing over the eye	82	26.7%
	A white spot in the eye	29	9.4%
	I don’t know	26	8.5%
	Cataracts decrease vision	238	77.5%
	Cataracts cause blindness	176	57.3%
	It is possible to get back vision from cataract blindness	208	67.8%
Risk factors of cataracts	Prevention of risk factors is possible	204	66.4%
	Hypertension	198	64.5%
	Old age	197	64.2%
	Diabetes mellitus	192	62.5%
	Trauma	189	61.6%
	Ultraviolet light	186	60.6%
	Obesity	181	59.0%
	Eye surgery	169	55.0%
	Refractive errors	162	52.8%
Treatment of cataracts	Treatment of cataract		
	Surgery	157	51.1%
	Medications	150	48.9%
	It is necessary to implant a lens in cataract surgery		
	Yes	187	60.9%
	No	120	39.1%

**Figure 1:**
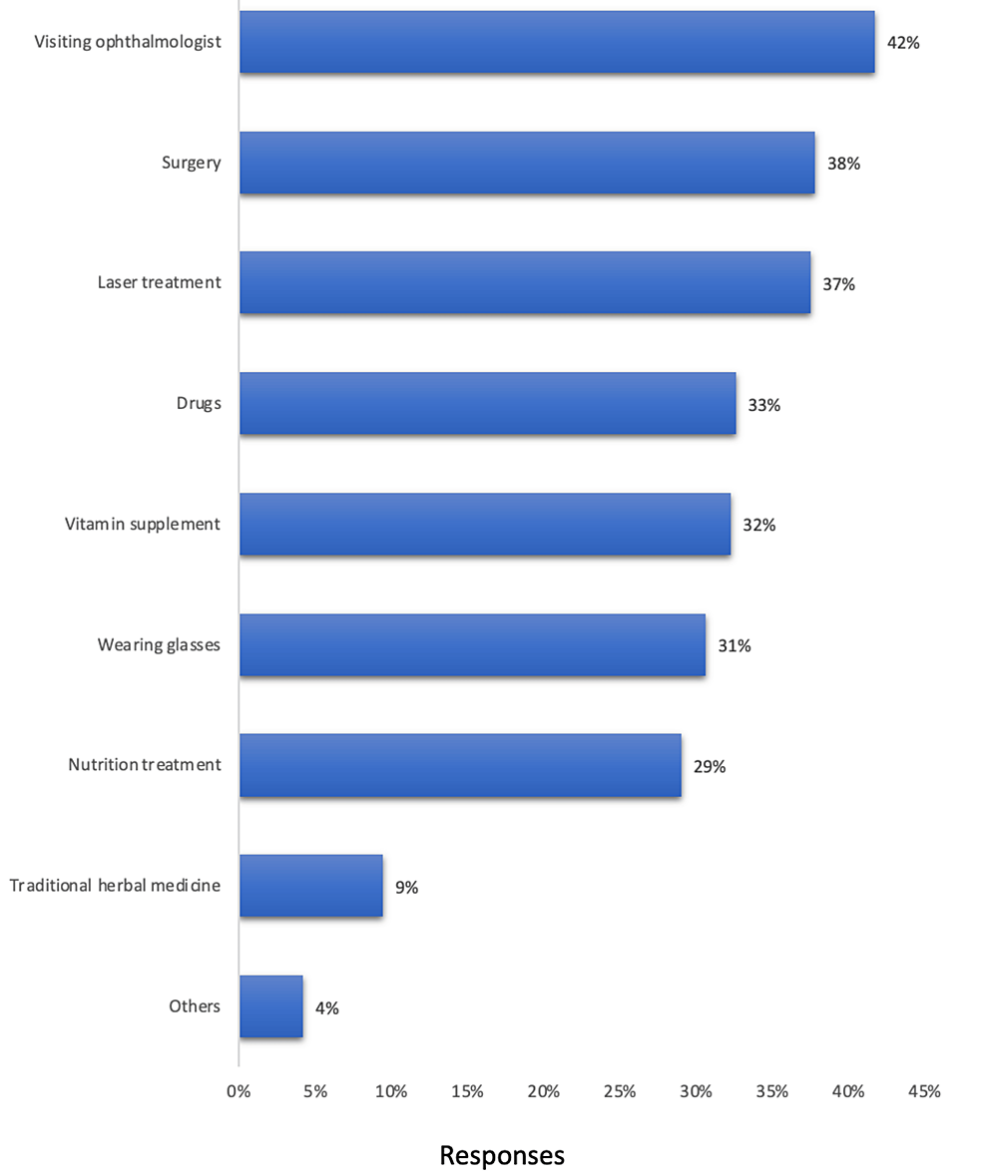
Cataract prevention methods reported by study participants, Hail, Saudi Arabia.

The most reported prevention methods were visiting an ophthalmologist (42%), surgery (38%), laser treatment (37%), and drugs (33%). The least known methods were traditional herbal medicine (9%) and others (4%) (Figure 1).

**Figure 2:**
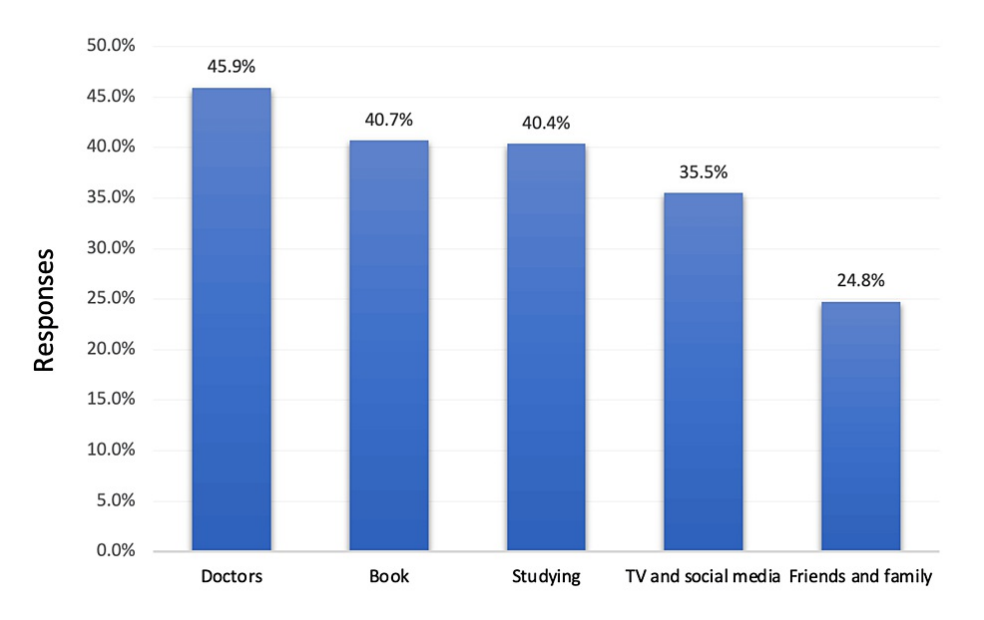
Source of information about cataracts and consequences among adults, Hail, Saudi Arabia.

The most reported source of information among study participants was doctors (45.9%) (Figure 2).

As shown in Table 3, 84 (27.4%) study participants agreed that a person with a cataract should seek eye treatment, 83 (27%) agreed that cataract is a serious health problem, 26.1% agreed that a person with a cataract needs an eye examination, and, surprisingly, 215 (70.0%) disagreed. The majority of participants, 219 (71.3%), had negative attitudes and awareness regarding cataracts.

**Table 3:** Participants’ attitudes and awareness regarding cataracts, Hail, Saudi Arabia.

Attitude items	Disagree		Neutral		Agree	
	N	%	N	%	N	%
How much do you agree that a person with cataracts needs an eye examination?	215	70.0%	12	3.9%	80	26.1%
How much do you agree that a person with cataracts should seek eye treatment?	193	62.9%	30	9.8%	84	27.4%
How much do you agree that a cataract is a serious health problem?	167	54.4%	57	18.6%	83	27.0%
How much do you agree that a person with cataracts gets appropriate care in a healthcare institution?	165	53.7%	45	14.7%	97	31.6%
How much do you agree that if you did not go for treatment it was because the doctor advised that immediate treatment is not required?	224	73.0%	33	10.7%	50	16.3%
Attitude	Negative			219 (71.3%)		
	Positive			88 (28.7%)		

**Figure 3:**
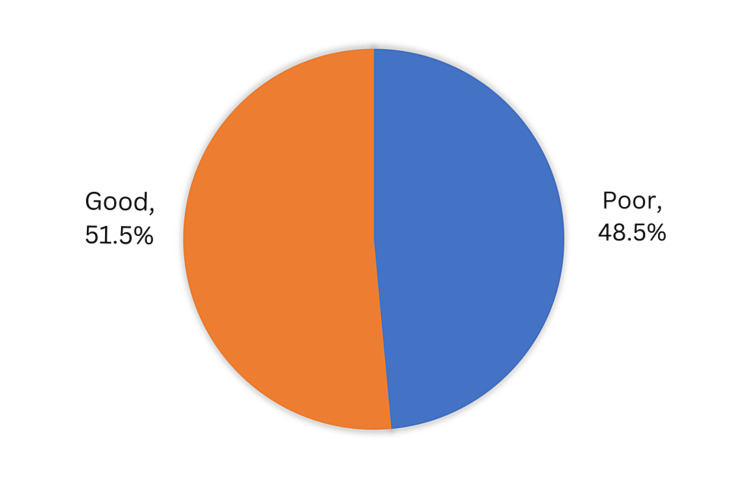
Participants’ knowledge regarding cataracts, consequences, risk factors, and treatment, Hail, Saudi Arabia.

In total, 158 (51.5%) participants had good knowledge of cataracts and related factors, while 149 (48.5%) had a poor knowledge level (Figure 3).

As shown in Table 4, 75% of participants aged 50 years or older had an excellent overall cataract knowledge level compared to 38.9% of others aged 30-39 years (p = 0.002). Moreover, 64.7% of unemployed/retired participants had a good knowledge level compared to 30% of healthcare staff (p = 0.007). Furthermore, 58.6% of participants with no cataract history had good knowledge versus 44.5% of others (p = 0.014).

**Table 4:** Distribution of participants’ knowledge of cataracts by their related factors and source of information. P: Pearson chi-square test; $: exact probability test; *: p < 0.05 (significant).

Factors	Knowledge level				P-value
	Poor		Good		
	N	%	N	%	
Age in years					0.002*^$^
20–29	46	39.3%	71	60.7%	
30–39	77	61.1%	49	38.9%	
40–49	23	44.2%	29	55.8%	
50+	3	25.0%	9	75.0%	
Gender					0.642
Male	69	50.0%	69	50.0%	
Female	80	47.3%	89	52.7%	
Marital status					0.276
Single	69	45.4%	83	54.6%	
Married	80	51.6%	75	48.4%	
Educational level					0.808
Secondary/Below	48	51.1%	46	48.9%	
University	88	47.1%	99	52.9%	
Postgraduate	13	50.0%	13	50.0%	
Work					0.007*
Not working/Retired	6	35.3%	11	64.7%	
Student	44	40.0%	66	60.0%	
Non-healthcare staff	71	50.7%	69	49.3%	
Healthcare staff	28	70.0%	12	30.0%	
Complain of cataract					0.014*
Yes	86	55.5%	69	44.5%	
No	63	41.4%	89	58.6%	
Source of information					0.253
TV and social media	47	43.1%	62	56.9%	
Book	62	49.6%	63	50.4%	
Doctors	77	54.6%	64	45.4%	
Studying	59	47.6%	65	52.4%	
Friends and family	34	44.7%	42	55.3%	

As shown in Table 5, Approximately 18.9% of female participants agreed that a person with a cataract needs an eye examination versus 34.8% of male participants (p = 0.004). Furthermore, 26% of female participants agreed that a person with a cataract gets appropriate care in a healthcare institution compared to 38.4% of male participants (p = 0.047). 

**Table 5:** Distribution of participants’ attitudes toward cataracts by their gender. P: Pearson chi-square test; $: exact probability test; *: p < 0.05 (significant).

Attitude items	Gender				P-value
	Male		Female		
	N	%	N	%	
How much do you agree that a person with cataracts needs an eye examination?					0.004*^$^
Disagree	87	63.0%	128	75.7%	
Neutral	3	2.2%	9	5.3%	
Agree	48	34.8%	32	18.9%	
How much do you agree that a person with cataracts should seek eye treatment?					0.086^$^
Disagree	78	56.5%	115	68.0%	
Neutral	14	10.1%	16	9.5%	
Agree	46	33.3%	38	22.5%	
How much do you agree that a cataract is a serious health problem?					0.128
Disagree	68	49.3%	99	58.6%	
Neutral	25	18.1%	32	18.9%	
Agree	45	32.6%	38	22.5%	
How much do you agree that a person with cataracts gets appropriate care in a healthcare institution?					0.047*
Disagree	68	49.3%	97	57.4%	
Neutral	17	12.3%	28	16.6%	
Agree	53	38.4%	44	26.0%	
How much do you agree that if you did not go for treatment it was because the doctor advised that immediate treatment is not required?					0.217
Disagree	95	68.8%	129	76.3%	
Neutral	15	10.9%	18	10.7%	
Agree	28	20.3%	22	13.0%	

## Discussion

Study participants aged 20-29 years scored 60 points, with seven participants showing a good knowledge of cataracts, while those aged 30-39 years demonstrated the opposite. However, another study in southern Ethiopia showed that adults (≥40 years) were twice more likely to have good knowledge about cataracts than younger individuals [14]. Of the study respondents, most students had more knowledge about cataracts than healthcare staff. Despite that, our study included all students, including medical students, so it is possible that their percentages were high. The study showed that participants complained of poor cataract knowledge. To enhance awareness regarding cataracts, it is imperative to intensify health education efforts, explain the condition to patients, and conduct community-wide awareness campaigns.

Regarding the attitudes toward cataracts, 29% of the participants showed a positive attitude, which is slightly less compared to a study done in southern Ethiopia (37.9%) [15], with both showing a low positive attitude. Moreover, males had more positive attitudes than females.

On the contrary, two recent studies from Saudi Arabia about the attitudes toward diabetes mellitus, one among diabetic patients in Jazan [16] and another among university students in Jeddah [17], showed a highly positive attitude. We can speculate the difference between the attitudes toward cataracts and diabetes mellitus is because of two main reasons. First, diabetes is prevalent in the community, and the fact that having relatives with the same condition enhances an individual’s attitude and knowledge about the condition. Second, the large number of awareness campaigns that are implemented in public places and schools help improve attitudes. The importance of assessing attitude is because the study population’s positive attitude toward cataracts can raise awareness regarding the causes, prevention, and treatment of cataracts [14].

The majority of participants did not know the correct definition of a cataract, as in a study in Gondar town, northwest Ethiopia, with about 51% of participants answering incorrectly [18].

Most participants knew that cataracts reduce peripheral vision, while more than half stated that cataracts induce blindness. A previous study showed that participants knew that blurred vision was the most common symptom, while less than half of the participants knew that cataracts can lead to blindness [14]. The same study showed that more than half of the participants learned about the risk factors of cataracts, and more than half knew about surgical treatment and extraction of cataract lenses [14]. The result of our study and the comparative analysis are favorable regarding treatment awareness. In our study, about 50% agreed that surgery is the treatment for cataracts, which is consistent with two studies from Nepal [19] and Gondar town, northwest Ethiopia [18]. The percentage of those who agreed was 64% and 67%, respectively in these studies [18,19]. Compared to the awareness regarding other chronic diseases in the Hail region, when asked about the treatment of glaucoma, some studies found that less than half of the participants selected eye drops to treat glaucoma, less than half chose surgery, and more than half chose both [20]. As a result, most of the participants had poor knowledge about glaucoma. Therefore, local health authorities must implement glaucoma awareness programs to change public perception and behavior. Another study that aimed to assess general knowledge in the Hail region found that regardless of education level, residence, or employment status, most participants (82%) had a high level of diabetes knowledge and were aware that physical activity can positively affect or prevent diabetes [21].

Limitations

Cataract is a diagnosis that largely affects the elderly population. Given that we collected our data using an online questionnaire which is harder for people with cataracts to fill out, it can be considered a limitation. If they were able to participate, they would have had better knowledge and understanding of cataracts.

## Conclusions

Cataracts are a preventable cause of blindness that can easily be corrected by surgery. In this study, we discovered extremely concerning rates of knowledge, awareness, and attitudes regarding cataracts among the population of Hail City. What is more concerning is the poor knowledge and understanding observed among participants diagnosed with cataracts. More educational programs should be directed toward cataracts for patients and the public.

## Appendices

Questionnaire

(A) Sociodemographic characteristics

1-   Gender:

·       Female

·       Male

2-   Age:

·       18-29 years old

·       30-39 years old

·       40-49 years old

·       Above 50

3-   Martial status:

·       Single

·       Married

4-   Educational level:

·       High school or less

·       College degree

·       Postgraduate

·       Uneducated

5-   Occupation:

·       Student

·       Teacher

·       Doctor

·       Nurse

·       Unemployed

·       Retired

·       Other

(B) Knowledge about cataracts

6-   Have you been diagnosed with cataract:

·       Yes

·       No

7-   What is cataract? 

·       A white membrane growing over the eye

·       A lens change where the lens becomes opaque

·       An age-related process leading to a decrease in vision 

·       A white spot in the eye

·       I don’t know

8-   Do cataracts decrease vision?

·       Yes

·       No

9-   Do cataracts cause blindness?

·       Yes

·       No

10-   Is it possible to get back vision from cataract blindness?

·       Yes

·       No

11-   Can age be a risk factor for cataracts?

·       Yes

·       No 

12-   Can trauma be a risk factor for cataracts?

·       Yes

·       No 

13-   Can UV light be a risk factor for cataracts?

·       Yes

·       No 

14-   Can eye surgery be a risk factor for cataracts?

·       Yes

·       No 

15-   Can vision problems (such as myopia) be a risk factor for cataracts?

·       Yes

·       No 

16-   Can hypertension be a risk factor for cataracts?

·       Yes

·       No 

17-   Can diabetes be a risk factor for cataracts?

·       Yes

·       No 

18-   Can obesity be a risk factor for cataracts?

·       Yes

·       No 

19-   Prevention of risk factors is possible?

·       Yes 

·       No 

20-   What is the mechanism of prevention of cataracts?

·      Wearing glasses

·      Surgery

·      Drugs 

·      Laser treatment

·      Vitamin supplement

·      Nutrition treatment 

·      Visiting ophthalmologist 

·      Traditional herbal medicine

·       Others

21-   How is cataract treated?

·       Medical 

·       Surgical 

22-   Is it necessary to implant a lens in cataract surgery?

·       Yes

·       No 

23-   Source of information (multiple choices)

·       TV and social media

·       Book

·       Doctors

·       Studying

·       Friends and family 

**Table 6:** Participant attitudes toward cataract

		Strongly disagree (1)	Disagree (2)	Neutral (3)	Agree (4)	Strongly agree (5)
	How much do you agree that a person with cataracts needs an eye examination?					
	How much do you agree that a person with cataracts should seek eye treatment?					
	How much do you agree that a cataract is a serious health problem?					
	How much do you agree that a person with cataracts gets appropriate care in a healthcare institution?					
	How much do you agree that if you did not go for treatment it is because of the doctor advising that immediate treatment is not required?					
